# CD4^+^CD25^+^ Regulatory T Cell Ontogeny and Preferential Migration to the Cecal Tonsils in Chickens

**DOI:** 10.1371/journal.pone.0033970

**Published:** 2012-03-27

**Authors:** Revathi Shanmugasundaram, Ramesh K. Selvaraj

**Affiliations:** Department of Animal Sciences, Ohio Agricultural Research and Development Center, Wooster, Ohio, United States of America; University Hospital Jena, Germany

## Abstract

Thymic CD4^+^CD25^+^ cells have regulatory-T-cell-like properties in chickens. This study examined the ontogeny of CD4^+^CD25^+^ cells in the thymus and in peripheral compartments in chickens. CD4^+^CD25^+^ cells started to appear in the thymus at day 15 of incubation (E15), although at low percentages. Expressed as a percentage of CD4^+^ cells, CD4^+^CD25^+^ cells increased (*P*<0.01) from 1.7% at E20 to 7.3% at 0 d post-hatch (D0). CD4^+^CD25^+^ cells did not appear in the spleen or cecal tonsils of embryos. Expressed as a percentage of CD4^+^ cells, CD4^+^CD25^+^ cells increased (*P*<0.01) from 0% at D0 to 27% at D1 in cecal tonsils and from 0% at D0 to 11% at D1 in the spleen. Expressed as a percentage of all mononuclear cells, cecal tonsils at D1 had approximately 3.5-fold higher percentage of CD4^+^CD25^+^ cells than the spleen at D1. CD4^+^CD25^+^ cells from cecal tonsils of chicks at D1 were suppressive. CD4^+^CD25^+^ cells from D0 thymus, when injected back into MHC-compatible chicks, migrated to cecal tonsils and lungs and were detected until 10 d post-injection. CD4^+^CD25^+^ cells from cecal tonsils had a higher (*P* = 0.01) relative amount of CCR9 mRNA than CD4^+^CD25^+^ cells from the thymus. It could be concluded that in chickens CD4^+^CD25^+^ cells migrate from the thymus immediately post-hatch and preferentially colonize the gut associated lymphoid tissues. CD4^+^CD25^+^ cells' preferential migration to cecal tonsils is likely directed through the CCR9 pathway in chickens.

## Introduction

Regulatory T cells (Tregs) are a subset of T cells involved in immune suppression. In mammals, Tregs are initially defined by the expression of CD4 and CD25 [1], though FoxP3 was later identified as a master regulator [2] and a specific marker for Tregs [3]. We previously described thymic CD4^+^CD25^+^ cells to have Treg-like properties in chickens, even though chickens lack FoxP3 [4]. In chickens, thymic CD4^+^CD25^+^ cells have the classical properties of Tregs, like high IL-10, TGF-β, cytotoxic T-lymphocyte antigen 4, and lymphocyte-activation gene 3 mRNA amounts, and they suppress naïve T cell proliferation *in vitro* [4]. In chickens, CD4^+^CD25^+^ cells are present in high numbers in mucosal regions like cecal tonsils and lungs. CD25, though is widely used as Treg marker, is not a Treg-specific marker and in periphery activated T cells can express CD25 transiently [5].

In mammals, Tregs develop in the thymus [6]. Initial export of Tregs from the thymus was originally proposed to occur between day 2 and 4, by which time conventional T cells have already migrated to the periphery [7]. This hypothesis is based on the observation that surgical neonatal thymectomy of normal mice, between day 2 and 4 after birth, led to autoimmune diseases [8] and that transferring Tregs prevents the induction of autoimmune diseases in d 3 thymectomized mice [1]. However, other studies have shown the presence of Tregs in the periphery of three-day-old mice [9] and, hence, it was later proposed that Tregs are exported from the thymus, along with conventional T cells, before birth [10]. In chicken embryos, the thymus is colonized in three distinct waves at 6.5, 12, and 18 days of incubation, and each wave is followed by export of lymphocytes [11], [12]. Export of lymphocytes from the thymus to colonize peripheral lymphoid organs also occurs in discrete waves [13].

The ontogeny of CD4^+^CD25^+^ cells in both the thymus and peripheral compartments in chickens has not been studied. In chickens [4] as well as in mammals [14], CD4^+^CD25^+^ Tregs preferentially migrate to the mucosal-associated lymphoid tissues. In mice, Treg migration to the mucosal-associated lymphoid tissue of the gut is directed through the chemokine receptor type 9 (CCR9) pathway [15]. The objectives of this study are to analyze the percentage of CD4^+^CD25^+^ cells in the thymic lobes, spleens, and cecal tonsils of developing embryos and of chicks post-hatch. In addition, the migration pattern of thymic CD4^+^CD25^+^ cells were studied. Thymic CD4^+^CD25^+^ cells were isolated and injected back into MHC-compatible chicks to identify the migration pattern of Tregs. The relative CCR9 mRNA amounts of CD4^+^CD25^+^ cells from different organs were analyzed to study the role of the CCR9 receptor on CD4^+^CD25^+^ cell trafficking to gut-associated lymphoid tissues.

## Materials and Methods

Tissues for experiments were collected from UCD 003, a White Leghorn inbred (99.9%) chicken with a defined MHC haplotype (B^17^/B^17^) maintained in specific pathogen free conditions. All chickens were maintained under standard animal husbandry conditions, fed standard chicken diets and raised in fresh wood-shaving litter. All animal protocols were approved by the Institutional Animal Care and Use Committee at The Ohio State University (2011A00000125).

### Anti-chicken CD25 monoclonal antibody

Production of mouse anti-chicken CD25 is described previously [4]. Briefly, the extracellular part of chicken CD25 protein was expressed. Mice were immunized against the partial chicken CD25 protein, and the splenic hybridomas were screened for anti-chicken CD25. The positive hybridoma clone was grown and the supernatant was concentrated for anti-CD25 antibodies as described previously [16]. The antibody concentration in the final solution was determined in a spectrophotometer to be 0.9 mg/ml.

### Percentage of CD4^+^CD25^+^ cells in different organs of developing embryos and post-hatch chicks

Fertile eggs were collected from approximately 60-week-old UCD 003 breeders and incubated under standard conditions. At d 15, 17, and 20 of incubation (referred to as E15, E17, and E20, respectively) and d 0, 1, 2, and 6 post-hatch (referred to as D0, D1, D2, and D6, respectively), thymic lobes, spleens, and rudimentary cecal tonsils were collected from nine embryos/chicks per time points. For each time point, organs from three individual embryos/chicks were pooled and pooled samples analyzed to yield three replicates (n = 3). For cecal tonsils, a 3 mm length of the intestine at the cecal-intestinal junction was excised. Single cell suspensions from the organs were enriched for mononuclear cells by density centrifugation over Histopaque (1.077 g/ml, Sigma Aldrich, St.Louis, MO). PE-linking of mouse anti-chicken CD25^+^ was conducted as described earlier [17]. Approximately 1×10^6^ cells were incubated with 10 µg/ml of PE-linked mouse anti-chicken CD25, 1∶200 FITC-conjugated mouse anti-chicken CD4 (Southern Biotech, Birmingham, AL), and 1∶200 dilution of unlabelled mouse IgG for 45 min in PBS buffer supplemented with 0.5% BSA and 2 mM EDTA. Unbound antibodies were removed by centrifugation at 400×g for 10 min and washed with PBS two times. The percentages of CD4^+^ and CD4^+^CD25^+^ cells were analyzed in a flow cytometer (Guava Eascyte, Millipore). Cells were gated based on forward and side scatter profiles to include mononuclear cells and exclude dead cells. The percentage of CD4^+^CD25^+^ cells was analyzed as a percentage of CD4^+^ cells and as a percentage of all cells in the sample.

### Suppressive properties of CD4^+^CD25^+^ cells from cecal tonsils of chicks at D1

CD4^+^CD25^+^ cells from cecal tonsils of chicks at D1 were sorted using the anti-PE multisort kit (#130-090-757, Miltenyi Biotech, Auburn, CA) following manufacturer's instructions. Cecal tonsils were collected from 18 chicks. Cecal tonsils from six individual chicks were pooled and pooled samples analyzed to yield three replicates (n = 3). Single cell suspensions from the cecal tonsils were enriched for mononuclear cells by density centrifugation. Lymphocytes (5×10^7^ cells) were incubated with 20 µg of PE-conjugated anti-chicken CD25 for 45 min and 1∶200 dilution of unlabelled mouse IgG in 1 ml of PBS buffer supplemented with 0.5% BSA and 2 mM EDTA (buffer). The unbound antibodies were removed by centrifugation, washed with PBS two times and incubated with 10 µl of anti-PE multisort PE beads in 100 µl of buffer for 15 min. The unbound beads were removed by centrifugation, and CD25^+^ cells were collected by positive selection in an MS column (Miltenyi Biotech, Auburn, CA) following manufacturer's instructions. The multisort beads were removed using the multisort release reagent (Miltenyi Biotech, Auburn, CA) following manufacturer's instructions. The CD25^+^ cells were incubated with 10 µg of APC-conjugated anti-chicken CD4 for 10 min in 100 µl of buffer. The unbound antibodies were removed by centrifugation and incubated with 10 µl of anti-APC conjugated beads (# 130-090-855, Miltenyi Biotech, Auburn, CA). The unbound beads were removed by centrifugation, and CD4^+^CD25^+^ cells were collected by positive selection in an MS column (Miltenyi Biotech, Auburn, CA) following manufacturer's instructions. CD4^+^CD25^−^cells from the cecal tonsils of chicks at D1 were collected using the same procedure described for CD4^+^CD25^+^ cells except that flow through cells, in the CD25^+^ cell retention step, were used for CD4^+^ positive selection to collect CD4^+^CD25^−^ cells. Purity of the collected CD4^+^CD25^+^ cells was 90, 90, 93% and the purity of the collected CD4^+^CD25^−^ cells was 94, 95 and 96% in the three replicates.

The suppression of T cell proliferation assay was employed to assess the suppressive properties of CD4^+^CD25^+^ and CD4^+^CD25^−^ cells from cecal tonsils. The suppression of T cell proliferation assay is a co-culture assay between CD4^+^CD25^+^ Tregs (effector cells) and carboxyfluorescein-succinimidyl-ester labeled (CFSE-labeled) CD4^+^CD25^−^ cells (responder cells), which has been described in detail previously [4], [17]. Naïve CD4^+^CD25^−^ T cells (responder cells) were collected from the spleen of one chicken, labeled with CFSE and stimulated *in vitro* with a 1∶750 dilution of soluble anti-chicken CD3 and CD28. A co-culture assay was performed by co-incubating 5×10^4^ CFSE-labeled responder cells with effector cells (CD4^+^CD25^+^ or CD4^+^CD25^−^ cells from cecal tonsils) at an effector∶responder cell ratio of 1∶1, or 0∶1 at 37°C in the presence of 5% CO_2_. The CFSE dilution of CFSE-labeled responder cells was measured at 72 h of co-culture as an indicator of cell proliferation (Guava Eascyte, Millipore). The unproliferated cell percentage in the co-culture group was determined after gating on the CFSE positive responder cells.

### *In vivo* migratory properties of CD4^+^CD25^+^ cells from the thymus of chicks at D0

Eighteen chicks at D0 were used for thymic CD4^+^CD25^+^ and CD4^+^CD25^−^ cell collection. Thymic lobes from six chicks were pooled to collect 3×10^7^ CD4^+^CD25^+^ and CD4^+^CD25^−^ cells as described above. Thymic CD4^+^CD25^+^ or CD4^+^CD25^−^ cells were labeled with 5 µM CFSE dye as described previously [18]. CFSE-labeled CD4^+^CD25^+^ or CD4^+^CD25^−^ cells (1×10^7^ cells per chick) were diluted in 500 µl PBS and injected intraperitoneally into four-week-old chicks of the same MHC haplotype (B17/B17) in three replications (n = 3) per time point. At 2, 5, and 10 d post-cell injection, spleens, cecal tonsils, and lungs were collected. Single cell suspensions from the spleens, cecal tonsils, and lungs (whole lobe) were enriched for mononuclear cells by density centrifugation and analyzed for CFSE positive cells using a flow cytometer (Guava Easycyte, Millipore) after gating on forward and side scatter.

### CCR9 mRNA content of CD4^+^CD25^+^ cells from the thymus, spleen, and cecal tonsils

Thymic lobes, spleens, and rudimentary cecal tonsils were collected from nine chicks per time points. For each time point, organs from three individual chicks were pooled and pooled samples analyzed to yield three replicates (n = 3). CD4^+^CD25^+^ cells were isolated from the thymus at D0 and D6, from the spleen at D2 and D6, and from cecal tonsils at D1 and D6 as described above. The purity of the CD4^+^CD25^+^ cells ranged from 89% to 92% in different organs. Total RNA was extracted using TRIzol reagent (Molecular Research Center, Cincinnati, OH) following manufacturer's instructions and analyzed for mRNA amounts of CCR9 (5′-gtgcctccctgagatcatgt-3′, 5′-tgtgcttttggcatcttttg-3′) [19], β-actin (5′-accggactgttaccaacacc-3′ and 5′-gactgctgctgacaccttca-3′) [4], and ubiquitin (5′-gggatgcagatcttcgtgaaa-3′ and 5′-cttgccagcaaagatcaacctt-3′) [20] by RT-PCR (iCycler, Biorad) using SyBr Green in duplicates. The annealing temperature for CCR9 primers was 56°C, β-actin was 57°C and ubiquitin was 55°C. The RT-PCR analysis has been described previously [17]. The relative mRNA content was normalized to the geomeans of β-actin and ubiquitin mRNA amounts as determined by the Normfinder program [21]. Fold change from the reference was calculated as 2^(Ct Sample-housekeeping)^/2^(Ct Reference-housekeeping)^, where Ct is the threshold cycle [22]. The reference group was defined as the CD4^+^CD25^+^ cells from the spleen at D2.

### Statistical analysis

A one-way ANOVA (JMP software, Cary, NC) was used to examine the effect of different parameters studied on dependent variables. When the effects were significant (*P*<0.05), differences between means were analyzed by Tukey's least square means comparison.

## Results

### Percentage of CD4^+^CD25^+^ cells in different organs of developing embryos and post-hatch chicks

**Figure 1:**
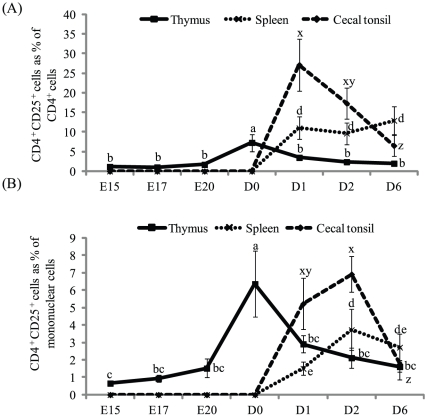
Percentage of CD4^+^CD25^+^ cells in different organs of developing embryos and post-hatch chicks. Fertile eggs were collected and incubated. At d 15 (E15), 17 (E17), and 20 (E20) of incubation and d 0 (D0), 1 (D1), 2 (D2), and 6 (D6) post-hatch, the thymic lobes, spleens and cecal tonsils were collected and analyzed for CD4^+^ and CD4^+^CD25^+^ cells. CD4^+^CD25^+^ cells were expressed either as a percentage of (A) CD4^+^ cells or (B) as a percentage of all mononuclear cells in the sample. a–c, Means (± SD) without a common superscript differ significantly in thymus (*P*<0.05). d–e, Means (± SD) without a common superscript differ significantly in spleen (*P*<0.05). x–z, Means (± SD) without a common superscript differ significantly in cecal tonsils (*P*<0.05). n = 3.

Percentages of CD4^+^CD25^+^ cells in the thymus, spleen, and cecal tonsils were dependent on the age of embryos or chicks (Fig. 1 and Fig. S1). CD4^+^CD25^+^ cells started to appear in the thymus at E15, although at low percentages. The percentage of CD4^+^CD25^+^ cells remained relatively low until D0, at which point the percentage of CD4^+^CD25^+^ cells increased (*P*<0.01). Expressed as a percentage of CD4^+^ cells, CD4^+^CD25^+^ cells increased from 1.7% at E20 to 7.3% at D0. CD4^+^CD25^+^ cells did not appear in the spleen or cecal tonsils of embryos or at D0. Expressed as a percentage of CD4^+^ cells, CD4^+^CD25^+^ cells increased from 0% at D0 to 27% at D1 in cecal tonsils (*P*<0.01) and from 0% at D0 to 11% at D1 in the spleen (*P*<0.01). Expressed as a percentage of all mononuclear cells, cecal tonsils had an approximately 3.5-fold higher percentage of CD4^+^CD25^+^ cells than the spleen at D1. Expressed as a percentage of CD4^+^ cells, the cecal tonsils and spleen had approximately the same percentage of CD4^+^CD25^+^ cells at D2 and D6. Expressed as a percentage of all cells, cecal tonsils had an approximately two-fold higher percentage of CD4^+^CD25^+^ cells than the spleen at D2.

### Suppressive properties of CD4^+^CD25^+^ cells from cecal tonsils of chicks at D1

**Figure 2:**
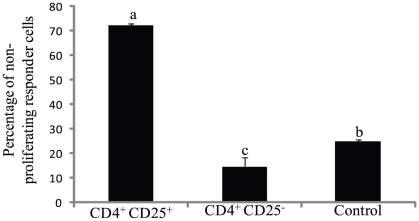
Suppressive properties of CD4^+^CD25^+^ cells from cecal tonsils of chicks at D1. Percentage of non-proliferating responder cells in proliferation suppression assay. CD4^+^CD25^−^ responder cells were labeled with carboxyfluorescein succinimidyl ester (CFSE), treated with anti-CD3/CD28, and mixed with effector CD4^+^CD25^+^ or CD4^+^CD25^−^ cells collected from cecal tonsils of one-day-old chicks at an effector∶responder cell ratio of 1∶1 or 0∶1 (control). At 72 h of co-culture CFSE dilution of CFSE-labeled responder cells was analyzed to calculate non-proliferating cell percentage. Means (+ SD) without a common superscript differ significantly (*P*<0.05). n = 3.

CD4^+^CD25^+^ cells from the cecal tonsils of chicks at D1 were suppressive (*P* = 0.01), whereas CD4^+^CD25^−^ cells from the cecal tonsils were not suppressive at an effector∶responder cell ratio of 1∶1 (Fig. 2 and Fig. S2). The percentage of non-proliferating CFSE-labeled responder cell in the CD4^+^CD25^+^ cell group was higher (*P*<0.01) than that in the CD4^+^CD25^−^ cell and the control group.

### *In vivo* migratory properties of CD4^+^CD25^+^ cells from the thymus of chicks at D0

**Figure 3:**
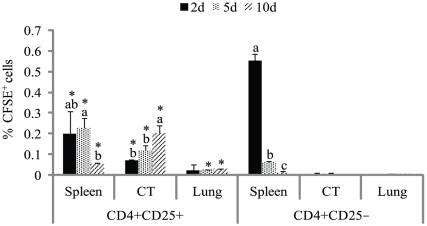
*In vivo* migratory properties of CD4^+^CD25^+^ cells from the thymus of chicks at D0. CD4^+^CD25^+^ or CD4^+^CD25^−^ cells were collected from the thymus of zero-day-old (D0) chicks, labeled with carboxyfluorescein succinimidyl ester (CFSE), and injected into MHC-compatible chicks. At d 2, 5, and 10 post-injection, the spleen, cecal tonsils (CT), and lungs were analyzed for CFSE^+^ cells. a–c, Means (± SD) without a common superscript differ significantly within an organ (*P*<0.05). * indicates significant differences (*P*<0.05) between CFSE^+^ cells in the CD4^+^CD25^+^ and CD4^+^CD25^−^ cells injected groups on a particular day. n = 3.

Migration patterns of CD4^+^CD25^+^ thymocytes were different from those of CD4^+^CD25^−^ cells (Fig. 3 and Fig. S3). At 2 d post-injection, though both injected CD4^+^CD25^+^ and CD4^+^CD25^−^ cells migrated to the spleen, injected CD4^+^CD25^−^ cell numbers were approximately three-fold higher (*P* = 0.01) than injected CD4^+^CD25^+^ cell numbers in the spleen. At 10 d post-injection, the percentage of injected CD4^+^CD25^+^ (*P* = 0.04) and CD4^+^CD25^−^ (*P* = 0.02) cells decreased in the spleen. Injected CD4^+^CD25^+^ cells migrated to the cecal tonsils and lungs at 2 d post-injection. The percentage of injected CD4^+^CD25^+^ cells increased (*P* = 0.01) in the cecal tonsils over time. The percentage of injected CD4^+^CD25^+^ cells was almost the same in the lungs over time (*P* = 0.94). Injected CD4^+^CD25^−^ cells did not migrate to the cecal tonsils or to the lungs at any of the time points studied.

### CCR9 mRNA content of CD4^+^CD25^+^ cells from the thymus, spleen, and cecal tonsils

**Figure 4:**
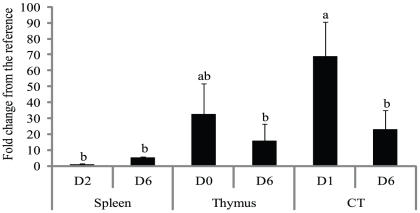
CCR9 mRNA content of CD4^+^CD25^+^ cells from the thymus, spleen, and cecal tonsils. CD4^+^CD25^+^ cells from the spleen at 2 d (D2) and 6 d (D6) post-hatch, the thymus at 0 d (D0) and 6 d (D6) post-hatch, and the cecal tonsils (CT) at 1 d (D1) and 6 d (D6) post-hatch were collected. The relative mRNA content was corrected for β-actin and ubiquitin mRNA amounts and normalized to the mRNA content of the spleen at D2, so that all bars represent fold change compared to the spleen at D2. Bars (means+SD) with no common superscript differ significantly (*P*<0.05). n = 3.

CD4^+^CD25^+^ cells from the spleen had the lowest relative amount of CCR9 mRNA at D2 and D6 (Fig. 4). CD4^+^CD25^+^ cells from cecal tonsils had a higher (*P* = 0.01) relative amount of CCR9 mRNA than the thymus at D6.

## Discussion

The ontogeny of CD4^+^CD25^+^ Treg-like cells in chickens was studied. CD4^+^CD25^+^ cells started to appear in the thymus at E15 at low percentages and remained relatively low until D0. At D0, the percentage of CD4^+^CD25^+^ cells in the thymus increased. The first export wave of thymic CD4^+^CD25^+^ cells migrated preferentially to the cecal tonsils, though a relatively small percentage of CD4^+^CD25^+^ cells migrated to the spleen. Later waves of CD4^+^CD25^+^ cells migrated to the spleen. Thymic CD4^+^CD25^+^ and CD4^+^CD25^−^ cells from D0 chicks, though migrated to the spleen at 2 d post-injection, only CD4^+^CD25^+^ cells were detectable in the cecal tonsils until 10 d post-injection. CD4^+^CD25^+^ cells from the cecal tonsils had the highest amount of CCR9 mRNA.

The percentage of both CD4^+^ and CD4^+^CD25^+^ cells changed during the study period in different organs and hence the percentage of CD4^+^CD25^+^ cells was expressed as both as a percentage of CD4^+^ cells and as a percentage of all cells in the sample. A very small percentage of CD4^+^ cells expressed CD25 before hatch. The percentage of CD4^+^CD25^+^ cells in the thymus increased several fold within 24 h of hatch. Colonization of the thymus by progenitor cells, differentiation of progenitors into a discrete lymphocyte subpopulation, and export of different subsets of lymphocytes out of the thymus occur in a series of waves in chickens [13], [23], [24]. At E12, 5 d after the first wave of thymus colonization, T cells with γ/δ TCR first appear in the thymus [25], and this subpopulation gets exported to peripheral organs as early as E17 [26]. The second wave of thymus colonization produces predominantly α/β TCR, and these T cells with α/β TCR emerge from the thymus at around E18 [27]. Because CD4^+^CD25^+^ cells did not appear in considerable numbers just before hatch, it is likely that progenitors that colonize the thymus in the third or later waves contribute to Tregs development. Waves of different subpopulations of lymphocytes that are exported from the thymus can alternate between different populations. For example, γ/δ and α/β cells migrate from the thymus in alternating waves [28]. Such export of cells from the thymus in waves can explain the increased production and export of CD4^+^CD25^+^ cells from the thymus between D0 and D1.

The early wave of CD4^+^CD25^+^ Treg-like cells from the thymus preferentially colonized the cecal tonsils. In chickens, CD25 is not a Treg-specific marker in the periphery, and activated T cells can upregulate CD25. However, such non-Tregs contributing to the CD25^+^ cell population in cecal tonsils is unlikely as CD4^+^CD25^+^ cells from the cecal tonsil had suppressive properties and high IL-10 mRNA content (data not shown). Non-Tregs with transient CD25 upregulation are not suppressive [4], [29]. In addition, CD4^+^CD25^+^ cells from D0 thymus, when injected intraperitoneally into four-week-old MHC-compatible chicks, migrated preferentially to the cecal tonsils. Four-week-old chickens were chosen because these layer chickens will be relatively small; hence, it was easy to identify injected cells. Further, layer chickens do not grow significantly during the 10-day experimental period. Though both the injected CD4^+^CD25^+^ and CD4^+^CD25^−^ cells were detected in the spleen at 2 d post-injection, only CD4^+^CD25^+^ cells were detected at 10 d post-injection. Cells injected through the peritoneal cavity have to leave the peritoneal cavity through the blood and lymph before they are distributed to the various tissues [30] the first of which is the parathymic lymph nodes in mammals [31]. Because chickens lack lymph nodes, injected cells might migrate to spleen. Surprisingly, a small percentage of injected CD4^+^CD25^+^ cells migrated to the lungs, another mucosal tissue in the body. In mammals CCR9-and its ligand-mediated interactions direct the migration of cells to mucosal tissue [32]. There is a possibility that the injected cells proliferated and lost the CFSE stain so that the injected cells were not detected.

Cecal tonsils are rudimentary in chicken embryos and at hatch have only low levels of lymphocytes. A major wave of lymphocyte colonization occurs at D4 [33]. At hatch, the cecal tonsils and spleen had negligible amounts of both CD4^+^ and CD4^+^CD25^+^ cells. At D1, both cecal tonsils and spleen had a low percentage of CD4^+^ cells. The proportion of CD4^+^CD25^+^ cells in the CD4^+^ population was higher in the cecal tonsils than in the spleen at D1. The higher proportion of CD4^+^CD25^+^ cells in the CD4^+^ population of the cecal tonsils than in the spleen is intriguing and could be related to the presence of gut microbes and development of the intestine immediately post-hatch. Post-hatch, the intestine, stimulated by food particles and gut microbes, undergoes extensive changes. In any organ, immune response is a balance between Tregs and T effector cells. Hyperactive Tregs can impair T cells, B cells, and other immune cell activity and, therefore, are implicated in impaired microbial defense and pathogen persistence [34]. Interestingly, we earlier reported that delaying duckling access to feed increases the CD25^+^ population and IL-10 mRNA content in cecal tonsils [35]. It could be hypothesized that Tregs, from early waves of thymic export, colonize the intestine, and exposure to feed and gut microbes will act as a feedback to decrease colonization of Tregs and to increase the number of T effector cells in the intestine. The number of CD4^+^ cells increased after D1 (Fig. S1).

CCR9 is a gut-homing receptor in T cells of chickens [19]. In mice, CCR9 directs the migration of Tregs to the lamina propria of the small intestine [36], and CCR9-deficient mice had impaired migration of Tregs to the gut mucosa [15]. The higher amount of CCR9 mRNA of the CD4^+^CD25^+^ cells from the cecal tonsils compared to the spleen suggests a direct role of CCR9 in CD4^+^CD25^+^ Treg-like cell migration to the gut or gut-associated lymphoid tissue.

In conclusion, CD4^+^CD25^+^ cells started to appear in the thymus at E15, although at low percentages that remained relatively low until D0, at which point the percentage of CD4^+^CD25^+^ cells increased. CD4^+^CD25^+^ Treg-like cells preferentially colonized the cecal tonsils before colonizing the spleen. Preferential migration of CD4^+^CD25^+^ Treg-like cells to cecal tonsils is likely directed through the CCR9 pathway.

## Supporting Information

Figure S1 **Representative flow cytometry figures of CD4 and CD25 staining for CD4^+^CD25^+^ cell analysis.** Fertile eggs were collected and incubated. At d 15 (E15), 17 (E17), and 20 (E20) of incubation and d 0 (D0), 1 (D1), 2 (D2), and 6 (D6) post-hatch, the thymus, spleen, and cecal tonsils were collected. Cells were enriched for mononuclear cells by density centrifugation, gated based on forward and side scatter profiles (A) to include mononuclear cells and exclude dead cells and analyzed for CD4^+^ and CD4^+^CD25^+^ cells (B).
(EPS)

Figure S2 **Suppressive properties of CD4^+^CD25^+^ cells from cecal tonsils of chicks at D1.** Representative flow cytometry figures from proliferation suppression assay. (a) CD4^+^CD25^−^ responder cells were labeled with carboxyfluorescein succinimidyl ester (CFSE), treated with anti-CD3/CD28, and mixed with effector (b) CD4^+^CD25^+^ or (c) CD4^+^CD25^−^ cells collected from cecal tonsils of one-day-old (D1) chicks at an effector∶responder cell ratio of 1∶1 or (d) 0∶1. CFSE dilution of CFSE-labeled responder cells was measured at 72 h of co-culture. Gate 1 measures percentage of effector cells, gate 2 measures percentage of proliferating responder cells, and gate 3 measures percentage of non-proliferating responder cell in the co-culture. Cells were not stained with any red fluorescence.
(EPS)

Figure S3 **Representative flow cytometry figures for analyzing i** ***n vivo*** **migratory properties of CD4^+^CD25^+^ cells.** CD4^+^CD25^+^ or CD4^+^CD25^−^ cells were collected from the thymus of zero-day-old chicks, labeled with CFSE, and injected into MHC-compatible chicks. At d 2, 5, and 10 post-injection, the spleen, cecal tonsils (CT), and lungs were analyzed for CFSE^+^ cells. Cells were not stained with any red fluorescence.
(EPS)
